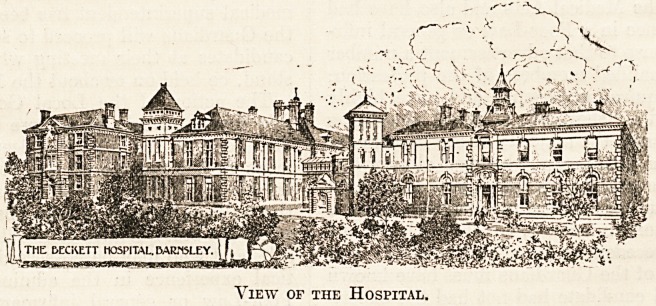# The Becket Hospital, Barnsley

**Published:** 1910-11-12

**Authors:** 


### THE BECKETT HOSPITAL, BARNSLEY.

### OPENING OF THE NEW WING.

CONSIDERABLE improvements and many alterations have been made at the Beckett Hospital, Barnsley, where on Thursday, October 27 last, Earl Fitzwilliam laid the foundation-stone of a new wing, which is the beginning of a well-thought-out extension scheme that practically remodels the whole institution, and will connect the various departments of the hospital into a comprehensive and easily worked plan. The hospital, it may be stated, was founded as a dispensary by Mr. John Beckett many years ago, but was soon remodelled and improved so as to include a series of three wards—Harvey, Sunderland, and Wentworth. Later on a new wing, known as the Kendray wing, was added, while a children's ward and Nurses Home were also built. Among the benefactors of the institution may be mentioned Mr. S. J. Cooper, who presented the Nurses' Home; Mr. William Moore, to whose generosity the hospital owes its convalescent home; Mr. Whitham, who inaugurated the fund that provided for the building of the children's ward; and Mr. Charles Harvey, whose gift was a good operating theatre.

### THE NEW BUILDINGS.

The present extension, which is to be called the Marshall wing, in memory of Mr. J. L. Marshall, of Monk Bretton, who bequeathed a special legacy to the hospital for starting the building fund, is really the completion of the Kendray block, and will provide thirty-six additional beds on two floors, and two single-bed wards. A sanitary spur on each floor is provided, with all modern conveniences and fittings, and separated from the wards by "cut-off" lobbies. In the high-pitch roof of this block accommodation is provided for seven maids' bedrooms, with separate fireproof staircase. Various enlargements and improvements will be made to the ward kitchens and bath-rooms in the Kendray block, and new linen-rooms and sink-rooms will be provided. The central or kitchen block is midway between the old and new buildings, and provides on the ground floor a new kitchen, with gas and steam cooking apparatus, scullery, maids' hall, etc., and ample storage, etc. In this block is also included a spacious sterilising room opening into the existing operating theatre. A new push-button electric lift is to be fitted, and all the existing hand-power lifts will be pulled out. This lift is a central feature of the scheme, and is placed on the main corridor. The first floor of this block provides accommodation for doctors' test-room, surgical stores, and a small steam-drying room. The alterations of the old wing consist of general re-arrangement and renovations to the women's wards, and to the various lavatories and rooms in connection therewith. The old kitchens have been converted into a recovery ward or short-period ward, to accommodate six beds, which will be used in connection with the out-patient and casualty department. The construction of floors, roofs, staircases, and ceilings will be of fireproof concrete. The floors generally will be finished in terazzo, though some of the floors will be in a patent jointless composition. The walls generally will be in patent plaster enamelled white. The kitchens, lavatories, larders, etc., will be lined with glazed tiles and bricks. The heating is to be by descending flue warm-air stoves, and a scheme for extending and centralising the hot-water apparatus is being considered. The contractors are as follows: Mason and brickwork, Messrs. W. G. and L. England, Limited; joiner's work, Messrs. J. Hawley and Sons, Penistone; slating, Messrs. Dawber, Townsley and Co.; plastering, Miss E. Fleming; plumbing, Mr. Edward Broley; architects, Messrs. Geo. Moxon and Son, Barnsley.

The cost of the scheme in hand is put at about £10,000, of which £7,073 has already been obtained.

### A SUGGESTION.

At the luncheon which followed the stone-laying ceremony Major Walker, according to the report in the *Barnsley Independent*, to which we are indebted for these particulars, brought forward an important suggestion and an appeal. He said it was a disgrace in a great mining district that working men who earned large wages—he had known one case where the earnings were more than £5 a week—should partake of all the benefits of science and skill at the hospital and not contribute anything towards it. It was taken by some as a matter of course, and never thought to be a duty to contribute something towards the cost of helping those who could not help themselves. He had spoken of this to the miners themselves, and he maintained that every man who received benefits from the hospital, and was in a position to give something, should contribute what his resources allowed. He believed in this district that people had got it into their heads that all those benefits could be got for nothing. He thought the committee might consider the matter and frame some inducement for his appeal to have effect.

In the course of the afternoon the following donations were announced: Captain Wentworth, £500; Earl Fitzwilliam, £500; Sir Joseph Walton, Bart., £100; Sir Hickman-Bacon, Bart., £25; Earl Crewe, £15; Bishop of Wakefield, £5. On the tables were cards and envelopes in which those present were asked to leave their donations, and it is understood the present result of the invitations amount to £1,700.

VIEW OF THE HOSPITAL.

## Figures and Tables

**Figure f1:**